# Rail‐tracking calcification of lower limb arteries

**DOI:** 10.1002/ccr3.1767

**Published:** 2018-08-14

**Authors:** Yoshito Kadoya, Kenji Yanishi, Satoaki Matoba

**Affiliations:** ^1^ Department of Cardiovascular Medicine Graduate School of Medical Science Kyoto Prefectural University of Medicine Kyoto Japan

**Keywords:** critical limb ischemia, Mönckeberg's arteriosclerosis, peripheral artery disease, rail‐tracking calcification

## Abstract

Peripheral artery disease has increased worldwide in recent years. Revascularization of severely calcified vessels is a technically challenging problem. Clinicians should recognize that excessive arterial calcification can occur even in nonelderly patients with less arteriosclerosis risk, leading to severe lower limb ischemia.

A 57‐year‐old woman presented with an 18‐year history of intermittent claudication and a 5‐month history of pain in both legs. There was no history of diabetes mellitus, chronic kidney disease, hypertension, hyperlipidemia, smoking, or abnormal bone morphology. Plain radiographs of bilateral lower limbs showed continuous, regular, tubelike calcification, described as “rail‐tracking calcification,” of the femoropopliteal arteries (Figure 1A). A peripheral angiogram revealed total occlusion of bilateral femoral arteries and development of numerous collateral arteries (Figure 1B). The patient was diagnosed with critical limb ischemia and suspicion of Mönckeberg's arteriosclerosis. After the autologous bone marrow cell transplantation, the patient's symptoms resolved.

**Figure 1:**
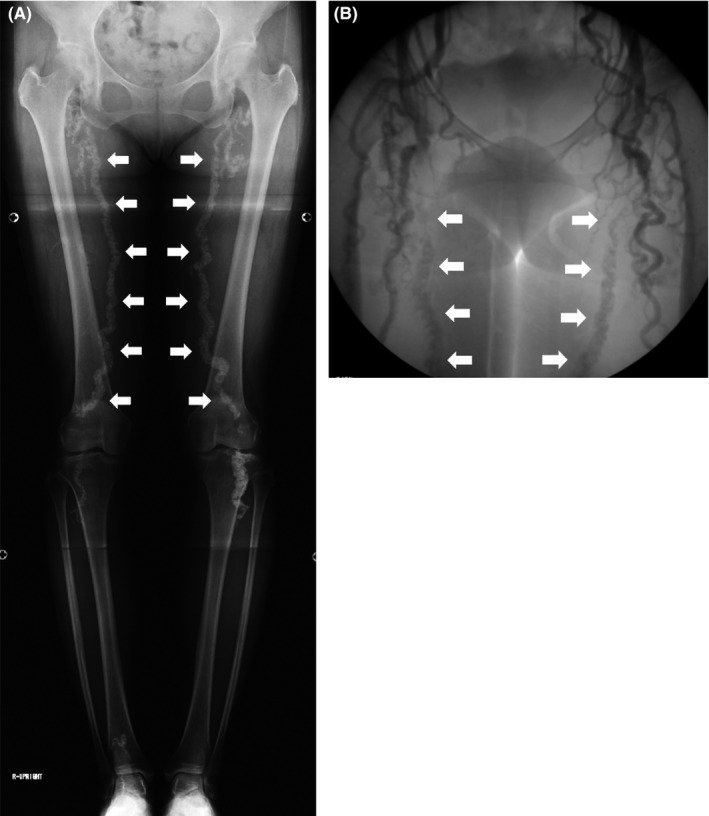
A, Plain radiographs of the lower limbs showing “rail‐tracking calcification” (arrows) of the femoropopliteal arteries bilaterally. B, Peripheral angiogram showing total occlusion of bilateral femoral arteries (arrows) and development of numerous collateral arteries

Peripheral artery disease (PAD) has increased worldwide in recent years. Revascularization of severely calcified vessels still remains a technically challenging problem. Mönckeberg's arteriosclerosis is a degenerative and noninflammatory disease characterized by dystrophic calcification of the tunica media of vessels due to the deposition of hydroxyapatite crystals.^1^ Continuous, regular, thin, tubelike calcification on plain radiographs suggests medial calcification.^2^ Recent research has shown that it can be an independent risk factor for cardiovascular disease and peripheral artery obstruction.^1^ Clinicians should recognize that excessive arterial calcification can occur even in nonelderly patients with less arteriosclerosis risk, leading to severe lower limb ischemia.

## CONFLICT OF INTEREST

None declared.

## AUTHORSHIP

YK: was involved in the clinical management of the case and manuscript redaction and correction. KY and SM: assisted in manuscript redaction and correction. All authors read and approved the final manuscript.
